# Simultaneous determination of l-tryptophan impurities in meat products

**DOI:** 10.1007/s00726-022-03215-8

**Published:** 2023-01-02

**Authors:** Doo-Hee Lee, Yang Hee Kim, Mina Baek, In Kyung Heo, Yonguk Shin

**Affiliations:** 1 National Instrumentation Center for Environmental Management, Seoul National University, Seoul, 08826 Republic of Korea; 2 Institute of Biotechnology, CJ CheilJedang, Suwon, Gyeonggi 16495 Republic of Korea

**Keywords:** l-tryptophan impurities, Eosinophilia-myalgia syndrome, LC–MS/MS, Meat products

## Abstract

L-tryptophan has been used as a feed additive for swine and poultry and as a nutrient supplement for humans. However, some impurities in l-tryptophan have been reported as causative components of eosinophilia-myalgia syndrome. Therefore, from a safety perspective, it is important to analyze meat samples for these impurities. This study aims to develop an analytical method for the simultaneous detection of l-tryptophan impurities in meat products using LC–MS/MS. Among the various impurities, detection methods for (S)-2-amino-3-(5-hydroxy-1H-indol-3-yl)propanoic acid (5-hydroxytryptophan) (HTP), 1-methyl-1,2,3,4-tetrahydro-β-carboline-3-carboxylic acid (MTCA), 3a-hydroxy-1,2,3,3a,8,8a-hexahydropyrrolo-[2,3-b]-indole-2-carboxylic acid (PIC), and 1,1′-ethylidenebistryptophan (EBT) and 2-(3-indoylmethyl)-l-tryptophan (IMT) were developed. The developed method allowed simultaneous determination of these four impurities in 5 min. No interferences from the matrix were observed, and the method showed good sensitivity to each analyte. The method detection limit and limit of quantification in meat matrices were below 11.2 and 35.7 μg/kg, respectively.

## Introduction

L-tryptophan ((S)-2-amino-3-(3-indolyl)propionic acid) is an essential amino acid in animal diets and is regarded as the fourth and third limiting amino acid for the growth of swine and poultry, respectively (Jansman et al. 2010; Peganova et al. 2003; Le Floc’h and Seve 2007). l-tryptophan is primarily used as a building block for protein synthesis. In addition to its effect on protein synthesis, l-tryptophan plays a role in other physiological processes that are associated with immune and inflammatory responses such as the synthesis of serotonin and niacin (Le Floc’h and Seve 2007; Sève 1999; Höglund et al. 2019; Fukuwatari and Shibata 2013).

Safety concerns regarding the use of l-tryptophan as a human dietary supplement have been raised because of its potential association with eosinophilia-myalgia syndrome (EMS). EMS is a rare condition characterized by inflammation in various organs, including the muscles, skin, and lungs. The clinical symptoms of EMS include subacute myalgia, eosinophilia in the peripheral blood, induration of skin, and chronic neuropathy; other common clinical signs include pneumonitis or pneumonia with or without pulmonary vasculitis, dyspnea, fatigue, rash, arthralgia, and edema of the limbs (Allen and Varga 2014; Aronson 2015). It has been reported that consumption of l-tryptophan human dietary supplement containing contamination caused EMS. The contamination occurred as a result of changes in the l-tryptophan manufacturing process at the Showa Denko K. K (SD) (Belongia et al. 1990; Philen et al. 1993; Blackburn 1997). The contamination was linked to the changes in the manufacturing process of SD l-tryptophan (Kilbourne 1992). When the SD l-tryptophan was analyzed using high-performance liquid chromatography (HPLC) and liquid chromatography–mass spectrometry (LC–MS), more than 60 impurities were found. Sample lot analyses and consequent epidemiological studies confirmed that 6 out of the 60 impurities were likely associated with the EMS outbreak in 1989: (1) 3-phenylaminoalanine (PAA), (2) 1,1′-ethylidenebistryptophan (EBT), (3) 2(3-indoylmethyl)-l-tryptophan (IMT), (4) 3a-hydroxy-1,2,3,3a,8,8a-hexahydropyrrolo-[2,3-b]-indole-2-carboxylic acid (PIC), (5) 2-(2-hydroxyindoline)-tryptophan (HIT), and (6) (S)-2-amino-3-(2-((S,E)-7-methylnon-1-en-1-yl)-1H-indol-3-yl) propanoic acid, or (S)-2-amino-3-(2-((E)-dec-1-en-1-yl)-1H-indol-3-yl) propanoic acid (AAA) (Mayeno et al. 1990; Hill et al. 1993; Williamson et al. 1998; Klarskov et al. 2018). In addition to these 6 compounds, two additional compounds that may be related to EMS, namely 1-methyl-1,2,3,4-tetrahydro-β-carboline-3-carboxylic acid (MTCA) and (S)-2-amino-3-(5-hydroxy-1H-indol-3-yl)propanoic acid (HTP), were mentioned (Das et al. 2004; Ogawa et al. 1993). HTP can be used as a substitute for l-tryptophan in physiological metabolism, and MTCA which is a decomposition product of EBT is expected as one of the EMS causative substances (Yamaguchi et al. 1991; Ito et al. 1992).

Methods for the analysis of l-tryptophan impurities, more specifically EMS-related substances, have been reported, and most are HPLC-based analyses. Simat et al. reported that the existence of these substances was analyzed from the pharmaceutical and feed grade of l-tryptophan by using HPLC (Simat et al. 2003). They provided the analytical method for MTCA, EBT, and IMT by using HPLC with a UV detector, of PAA by using HPLC with an amperometric detector, and PIC by using HPLC with a fluorescence detector. The detection limit of the method was between 0.5 and 7 mg/kg. Another research group reported an HPLC analysis method for the detection of MTCA, EBT, and PAA by using UV and fluorescence detection after separating the analyte with different cartridges (PRS, SCX, C8) (Adachi et al. 2000). Recently, the EBT analysis method from dietary supplements containing l-tryptophan was reported based on LC–MS/MS (Karakawa et al. 2022).

The l-tryptophan impurities in feed-grade l-tryptophan lead to human consumption through meat products. Therefore, the potential human intake of l-tryptophan impurities by consuming meat products should be considered. In this study, a simultaneous analytical method of l-tryptophan impurities in meat products was developed using LC–MS/MS that showed higher sensitivity than the previously reported methods.

## Materials and methods

### Materials

(S)-2-Amino-3-(5-hydroxy-1H-indol-3-yl)propanoic acid (5-hydroxytryptophan) (HTP) and 1-methyl-1,2,3,4-tetrahydro-β-carboline-3-carboxylic acid (MTCA) were procured from Sigma Aldrich (St. Louis USA). 3a-Hydroxy-1,2,3,3a,8,8a-hexahydropyrrolo-[2,3-b]-indole-2-carboxylic acid (PIC), 1,1′-ethylidenebistryptophan (EBT) and 2-(3-indoylmethyl)-l-tryptophan (IMT) were procured from Wuxi Apptec Co., Ltd. (Tianjin, China), Carbosynth (Berkshire, UK), and TLC Pharmaceutical Standards (Ontario, Canada), respectively.

All reagents used in the analysis were of LC–MS grade and used without further purification. Ammonium formate (≥ 99% purity) and formic acid (≥ 98% purity) were procured from Sigma Aldrich (St. Louis, USA). Methanol (100% purity) and water were procured from Merck (Darmstadt, Germany).

Meat samples (pig and chicken muscle) originating from Korea, Spain, and United States were collected from a local supermarket in Korea. And other meat samples (pig muscle, skin and organs) were obtained from a slaughterhouse in Korea. The samples were stored at − 20 °C until analysis.

### Sample preparation

The samples of pig muscle, pig skin, pig liver, pig kidney, pig lung, pig serum, and chicken muscle used for the spiking test and the commercial meat product were homogenized by grinding using a blender.

To obtain spiked samples, an appropriate amount of each standard chemical was added to the homogenized meat samples. The homogenized samples (1 g) were placed into a conical tube. Then, 80% methanol (10 mL) was added to the tube. The mixture was shaken and sonicated for 1 h. After sonication, the samples were centrifuged at 15,000 rpm for 10 min. The supernatants were taken and filtered using a 0.2 μm PTFE syringe filter.

### Analytical method

Chromatographic separation of the samples was performed on a UHPLC Vanquish (Thermo Scientific, USA) system using a Cortecs C8 (2.1 mm × 150 mm, 1.6 μm) (Waters, USA). The column oven was operated at 45 °C. Mobile phase A consisted of 10 mM ammonium formate and 0.1% formic acid in the water, and mobile phase B consisted of 0.1% formic acid in methanol. An optimized gradient elution with mobile phase A and mobile phase B (0–0.1 min, 10% B, 0.35 mL/min; 0.1–2.0 min, 30% B, 0.35 mL/min; 2.0–3.5 min, 100% B, 0.35 mL/min; 3.5–5.0 min, 100% B, 0.35 mL/min; 5.0–7.0 min, 10% B, 0.4 mL/min) were used.

A TSQ Altis triple quadrupole mass spectrometer (Thermo Scientific, USA) equipped with a heated electrospray ionization (H-ESI) ion source was used. This instrument was coupled to the UHPLC system. The source parameters of optimized mass spectrometry were positive ion 3500 V, sheath gas 50 Arb, aux gas 10 Arb, sweep gas 1 Arb, ion transfer tube temperature 325 °C, and vaporizer temperature 350 °C. Trace Finder 4.1 software (Thermo Scientific, USA) was used for the data analysis. Selected reaction monitoring (SRM) transitions were monitored to quantify the analytes. The SRM parameters are described in Table 1.

**Table 1:** Selected reaction monitoring (SRM) properties for the analysis of l-tryptophan impurities

Compound	Formula	Retention time (min)	Precursor (*m*/*z*)	Product (*m*/*z*)		Collision energy (V)	
				Quantifier ion	Qualifier ion	Quantifier ion	Qualifier ion
HTP	C_11_H_12_N_2_O_3_	1.25	221.1	162.1	134.1 204.2	18.9	26.1 11.0
PIC	C_11_H_12_N_2_O_3_	1.51	221.1	130.1	158.1 175.1	30.7	20.5 13.1
MTCA	C_13_H_14_N_2_O_2_	3.73	231.1	158.1	143.1 214.2	17.0	34.6 12.1
IMT	C_20_H_19_N_3_O_2_	4.62	334.2	130.1	205.2 217.2	20.8	11.4 11.1
EBT	C_24_H_26_N_4_O_4_	4.65	435.2	157.1	214.2 231.2	41.4	28.9 16.1

### Method performance evaluation

The analytical method developed in this study was validated by referring to official guidance (ICH 2005; EC 2002, 2021). The performance of the analytical method was evaluated considering the specificity, method detection limit (MDL), limit of quantification (LOQ), interday precision, recovery, and matrix effect.

The linearity of the method was evaluated with calibration curves prepared in matrices by spiking analytes in pig muscle, pig skin, pig liver, pig kidney, pig lung, pig serum, and chicken muscle. The regression equation and coefficient of each calibration curve were calculated.

The method detection limit and limit of quantitation were determined by analyzing seven samples with concentrations near the expected limit of detection. The standard deviation of seven samples was simply multiplied by the correct Student’s *t *value. The *t *value for six degrees of freedom and a 99% confidence level is 3.14. Therefore, the MDL and LOQ were calculated to be 3.14 × sd (standard deviation) and 10 × sd (standard deviation), respectively.

The interday precision was determined using a meat matrix spiked with analytes at the levels of 20 ng/mL. It was determined by analyzing the sample on seven consecutive days.

Recovery was evaluated at three fortified levels of analytes within the linear range of the calibration curve. The percentage of recovery was calculated as the concentration determined by the following equation:

$${\text{Recovery }}\left( \% \right) \, = { 1}00 \times {\text{measured concentration}}/{\text{fortified concentration}}.$$

The matrix effects of the analysis were assessed by comparing the peak area of the standards in solvent and matrix. It was evaluated according to the following equation:

$${\text{Matrix effect }}\left( \% \right) \, = { 1}00 \times {\text{peak area }}\left( {{\text{matrix}}} \right)/{\text{peak area }}\left( {{\text{solvent}}} \right) - {1}00.$$

## Results and discussion

### Specificity

**Fig. 1:**
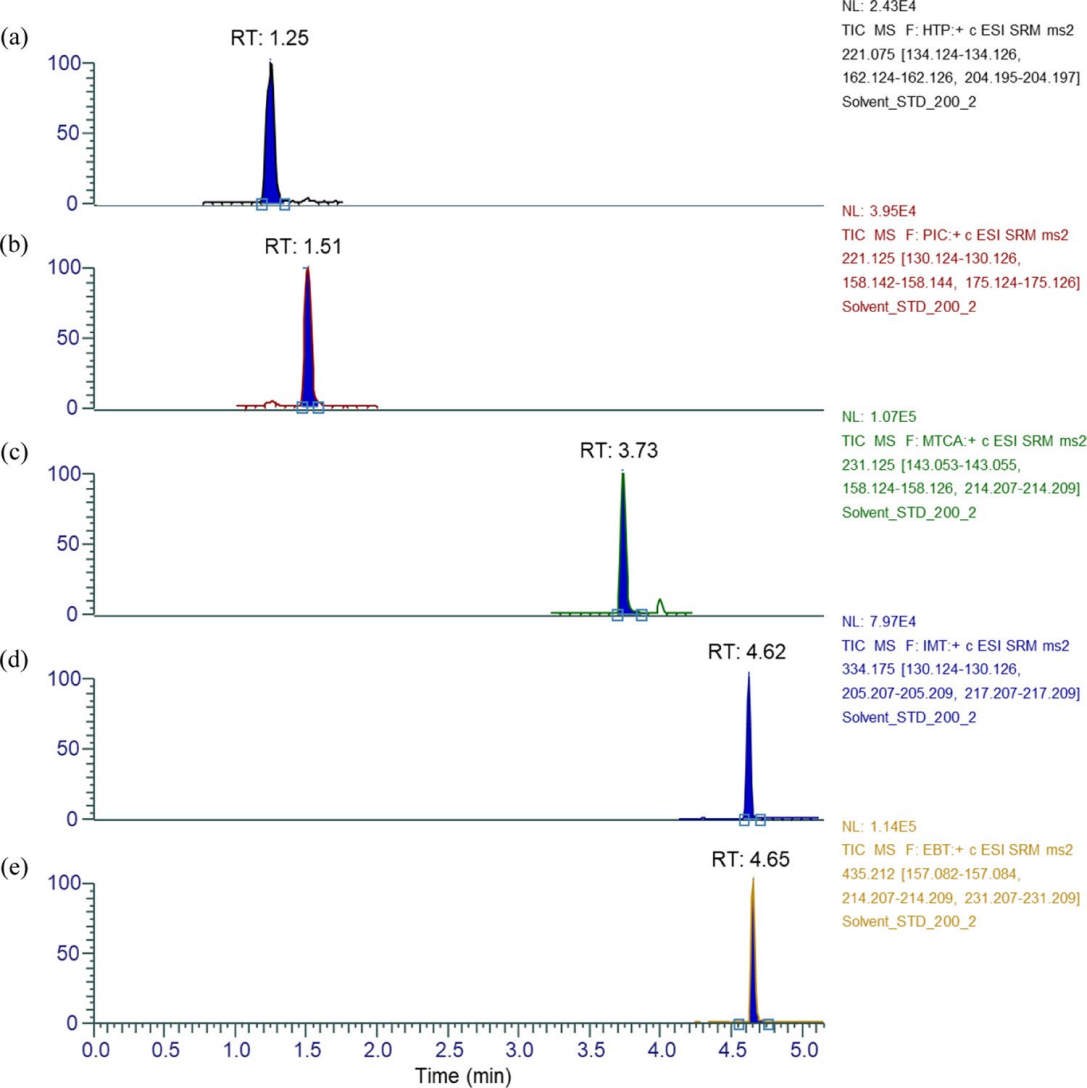
LC–MS/MS (SRM) chromatogram of l-tryptophan impurities **a** 5-hydroxytryptophan, **b** 3a-hydroxy-1,2,3,3a,8,8a-hexahydropyrrolo-[2,3-b]-indole-2-carboxylic acid, **c** 1-methyl-1,2,3,4-tetrahydro-β-carboline-3-carboxylic acid, **d** 2-(3-indoylmethyl)-l-tryptophan, and **e** 1,1'-ethylidenebistryptophan standards at 200 μg/mL

The chromatogram of the compounds is shown in Fig. 1. No significant interfering peak in the LC–MS/MS chromatogram was observed under the developed analysis conditions. Individual peaks for each analyte were separated well with a run time of less than 5 min.

### Linearity

The linearity of the analysis was observed through the calibration curves of each analyte obtained using the matrices spiked with the analytes. Linear regression analysis was conducted by plotting the peak area and concentration of the analyte. The linear range of the method is summarized in Table 2. The parameters of calibration for each analyte showed very good linearity, with a mean regression coefficient (*R*^2^) above 0.99.

**Table 2:** Linear range, coefficient of determination (*R*^2^), method detection limit (MDL), and limit of quantification (LOQ) for l-tryptophan impurities in meat matrices

Compound	Meat matrix	Linear range (ng/mL)	*R*^2^	MDL (μg/kg)	LOQ (μg/kg)
HTP	Pig muscle	2–50	1.0000	2.4	7.8
	Pig skin	2–50	1.0000	1.5	4.7
	Pig liver	2–50	0.9999	2.4	7.6
	Pig kidney	2–50	0.9999	2.9	9.4
	Pig lung	2–50	0.9990	3.0	9.4
	Pig serum	2–50	1.0000	1.2	3.7
	Chicken muscle	2–50	0.9998	3.5	11.1
PIC	Pig muscle	2–200	0.9999	1.5	4.9
	Pig skin	1–50	0.9999	2.0	6.4
	Pig liver	5–200	1.0000	1.9	6.2
	Pig kidney	5–200	0.9996	4.1	13.0
	Pig lung	10–200	0.9999	2.6	8.4
	Pig serum	1–100	0.9999	1.7	5.4
	Chicken muscle	2–100	0.9999	2.8	8.9
MTCA	Pig muscle	2–100	0.9999	1.1	3.5
	Pig skin	2–100	1.0000	2.3	7.5
	Pig liver	1–50	0.9999	4.3	13.7
	Pig kidney	1–50	1.0000	3.3	10.5
	Pig lung	2–50	1.0000	1.4	4.6
	Pig serum	1–100	1.0000	1.6	5.2
	Chicken muscle	1–50	1.0000	2.3	7.5
IMT	Pig muscle	2–100	1.0000	2.8	8.9
	Pig skin	1–50	0.9999	1.0	3.2
	Pig liver	1–50	0.9999	2.7	8.5
	Pig kidney	1–50	1.0000	2.4	7.8
	Pig lung	1–50	0.9998	2.3	7.3
	Pig serum	1–50	1.0000	3.0	9.5
	Chicken muscle	2–50	1.0000	2.7	8.7
EBT	Pig muscle	1–50	0.9999	2.3	7.4
	Pig skin	1–100	0.9997	2.3	7.4
	Pig liver	1–50	1.0000	11.2	35.7
	Pig kidney	1–50	1.0000	6.8	21.7
	Pig lung	1–50	0.9999	4.6	14.5
	Pig serum	1–50	0.9999	1.4	4.3
	Chicken muscle	2–50	1.0000	5.6	17.8

### Method detection limit and limit of quantification

The matrix-matched standard solutions of HTP, PIC, EBT, IMT, and MTCA were prepared by adding appropriate amounts of each compound to various meat matrices. The method detection limit (MDL) and limit of quantification (LOQ) of five analytes were calculated based on the seven samples of concentrations near the expected MDL (5 ng/mL). The MDL and LOQ of the analytes are summarized in Table 2. The MDL and LOQ of this analytical method were lower than 11.2 and 35.7 μg/kg, respectively. Although the analytical method developed to detect l-tryptophan impurities in meat products, it showed a lower detection limit than the previous data in less complex matrices such as a finished product of l-tryptophan and dietary supplement (Adachi et al. 2000; Simat et al. 2003; Karakawa et al. 2022).

### Accuracy and precision

The accuracy and precision of the method were assessed by a recovery test conducted by analyzing the known quantities of each compound added to the matrix-matched sample. As shown in Table 3, the accuracy was expressed by the recovery percentage, and precision was expressed by the relative standard deviation (% RSD).

**Table 3:** Recovery and relative standard deviation (RSD) of the l-tryptophan impurities in meat matrices at four fortified levels of 5, 10, 20, and 50 ng/mL

Compound	Fortified level (ng/mL)	Pig muscle		Pig skin		Pig liver		Pig kidney		Pig lung		Pig serum		Chicken muscle	
		Recovery (%)	RSD (%)	Recovery (%)	RSD (%)	Recovery (%)	RSD (%)	Recovery (%)	RSD (%)	Recovery (%)	RSD (%)	Recovery (%)	RSD (%)	Recovery (%)	RSD (%)
HTP	5	102.97	0.52	100.17	0.10	108.83	1.16	101.65	1.02	98.08	1.02	99.73	0.43	106.66	0.88
	10	105.02	1.77	103.79	1.63	105.37	0.81	102.00	1.00	105.24	1.31	100.86	1.73	100.91	0.48
	20	104.15	0.73	98.63	2.30	99.94	2.58	101.59	0.99	109.87	0.17	100.96	1.32	102.84	1.16
	50	98.32	0.60	105.73	1.30	98.86	1.13	101.45	0.25	108.55	2.64	98.75	0.93	97.74	0.79
PIC	5	105.49	0.70	105.33	0.42	99.69	0.84	108.87	2.09	90.77	2.70	99.92	1.24	110.19	1.58
	10	107.18	0.75	106.17	2.98	95.55	0.61	97.55	1.66	95.63	0.84	105.13	1.13	97.01	0.72
	20	112.73	0.46	107.72	1.39	97.98	1.71	96.99	2.57	92.91	1.93	102.58	2.17	99.05	0.73
	50	104.55	1.92	102.81	2.12	100.78	1.18	95.94	0.39	97.13	3.08	99.94	1.61	96.66	2.11
MTCA	5	106.62	0.31	101.39	0.36	98.33	1.08	101.74	0.83	99.19	0.62	100.43	1.03	99.40	1.69
	10	108.59	0.20	101.49	0.89	95.84	0.79	98.49	1.74	103.21	3.59	96.39	1.83	97.56	2.53
	20	92.62	0.10	97.43	1.39	93.77	0.89	93.55	2.01	99.47	1.25	99.22	1.31	99.67	3.61
	50	95.17	1.03	106.45	0.51	101.20	2.63	103.88	1.46	98.46	0.85	97.91	0.63	100.74	0.66
IMT	5	103.96	1.11	108.42	0.42	97.09	1.23	115.93	0.28	97.68	2.15	92.83	0.48	101.14	1.33
	10	108.26	0.59	103.79	2.16	101.33	1.28	104.91	2.36	106.45	1.53	92.10	1.83	102.55	3.24
	20	102.39	2.18	105.46	2.58	90.81	2.21	103.84	2.73	95.34	0.78	95.78	0.22	95.70	2.11
	50	105.69	1.32	110.20	1.96	99.16	0.54	100.66	2.30	102.26	0.64	98.16	1.02	98.37	0.83
EBT	5	110.65	0.74	107.44	1.10	98.43	2.78	99.99	1.67	96.13	1.48	99.79	0.96	99.84	1.31
	10	111.28	0.68	103.56	1.25	94.83	5.63	102.68	1.83	98.63	0.51	97.61	0.31	101.96	5.38
	20	107.57	1.02	107.17	0.26	97.34	4.93	89.28	0.59	96.61	4.63	97.38	2.40	96.31	4.45
	50	102.88	0.65	107.62	1.07	100.02	0.63	102.29	0.58	102.72	1.75	98.43	0.32	94.82	3.28

The matrix-matched sample was spiked with HTP, PIC, EBT, IMT, and MTCA at 4 different concentrations (5, 10, 20, 50 ng/mL). Table 3 presents the recovery and RSD of the test. The recovery of the analysis ranged from 92.6 to 112.7% for pig muscle, from 97.4 to 110.2% for pig skin, from 90.8 to 108.8% for pig liver, from 89.3 to 115.9% for pig kidney, from 90.8 to 109.9% for pig lung, from 98.8 to 105.1% for pig serum, and from 94.8 to 110.2% for chicken muscle.

For the interday precision, the test solution with the same concentration (20 ng/mL) was analyzed on seven consecutive days (Table 4). The interday precision ranged from 1.29 to 3.35% for pig muscle, from 1.32 to 3.77% for pig skin, from 2.26 to 4.55% for pig liver, from 1.65 to 4.44% for pig kidney, from 2.46 to 6.35% for pig lung, from 0.70 to 1.20% for pig serum, and from 1.39 to 4.96% for chicken muscle.

**Table 4:** Recovery and relative standard deviation (RSD) of the l-tryptophan impurities in meat matrices at a fortified level of 20 ng/mL (interday precision)

Compound	Fortified level (ng/mL)	Pig muscle		Pig skin		Pig liver		Pig kidney		Pig lung		Pig serum		Chicken muscle	
		Recovery (%)	RSD (%)	Recovery (%)	RSD (%)	Recovery (%)	RSD (%)	Recovery (%)	RSD (%)	Recovery (%)	RSD (%)	Recovery (%)	RSD (%)	Recovery (%)	RSD (%)
HTP	20	102.63	1.29	95.68	3.77	100.90	2.26	101.23	2.58	104.00	6.35	101.47	1.20	103.14	1.39
PIC	20	112.81	1.58	106.00	2.44	99.18	2.62	99.80	4.44	93.97	2.46	101.05	1.20	98.37	2.22
MTCA	20	100.99	3.25	99.78	1.32	93.70	4.18	92.82	2.38	94.29	3.38	96.90	0.77	99.10	3.04
IMT	20	103.53	3.35	107.42	1.72	88.14	4.55	102.86	1.65	94.64	4.11	94.11	0.92	92.99	3.68
EBT	20	106.86	3.05	109.78	1.89	101.38	3.10	85.87	4.45	95.09	4.88	97.52	0.70	95.05	4.96

Generally, the acceptable range of mean recoveries at the relevant concentration level should be 70–120% and the precision ≤ 20% RSD (EC 2021). For all other matrices, the range of mean recoveries at the fortified concentration level was 80–120%, and the precision was less than 10% RSD. Therefore, it was clear that the accuracy and precision of the proposed method for determining l-tryptophan impurities in the meat products were within the expected ranges indicating the high accuracy and precision of the method.

### Matrix effects

**Fig. 2:**
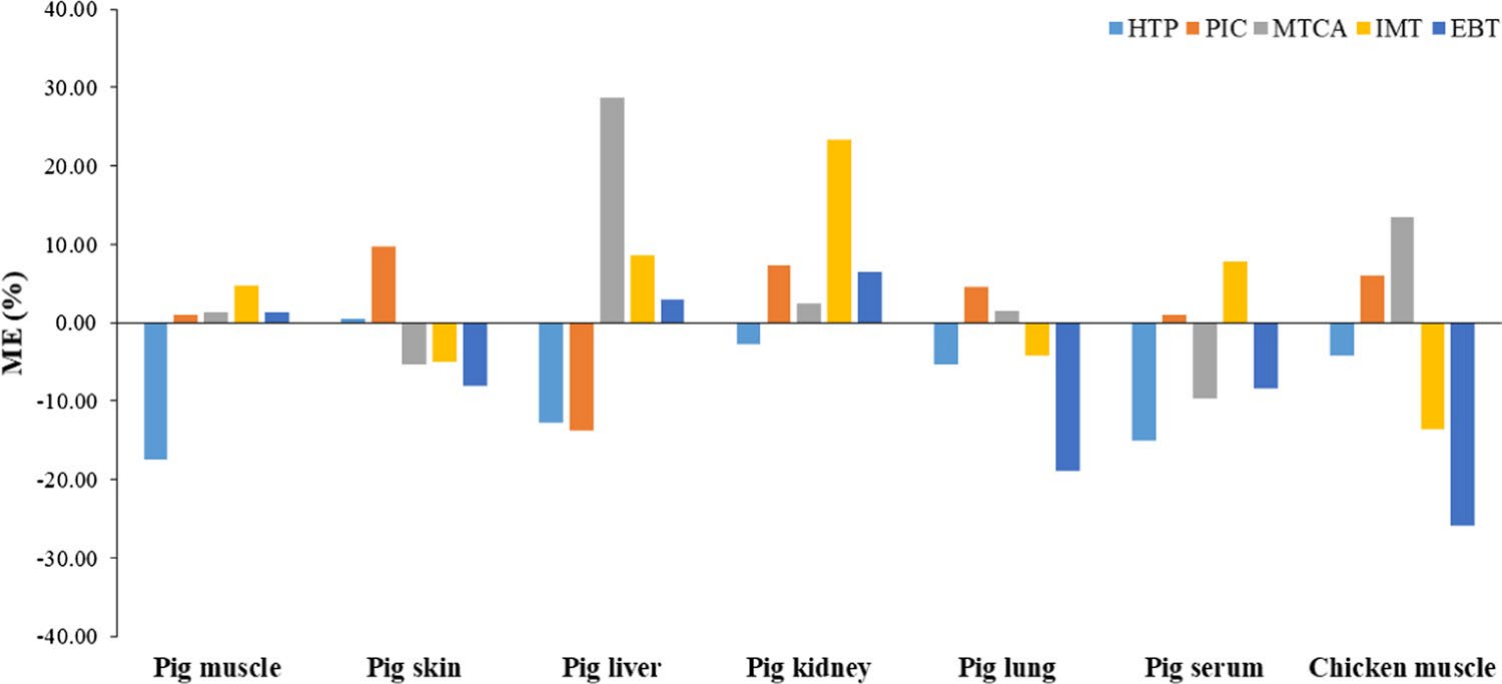
Matrix effects of the method for l-tryptophan impurities in seven food matrices (pig muscle, pig skin, pig liver, pig kidney, pig lung, pig serum, and chicken muscle). It was assessed by comparing the peak area of each standard in solvent and matrix

Matrix effect has been known as a major concern in quantitative LC–MS analysis because it affects the accuracy precision, and sensitive of the method (Zhou et al. 2017). Especially, a high concentration of nonvolatile materials solute in an extracted sample from the biological products caused the ionization suppression when ESI was used as an ionization technique (King et al. 2000). Due to the complexity of the meat matrix, the matrix effect of the developed method was evaluated by comparing the peak area of the solvent and the matrix-matched standard. The calculated matrix effects for each of the analytes in the meat matrices were summarized in Fig. 2. The percentage of the matrix effect ranged from − 17.4 to 4.7 for pig muscle, from − 8.1 to 9.7 for pig skin, from − 13.8 to 28.7 for pig liver, from − 2.8 to 23.4 for pig kidney, from − 19.0 to 4.6 for pig lung, from − 15.0 to 7.8 for pig serum, and from − 25.9 to 13.5 for chicken muscle.

Overall, the matrix effects ranged from − 20 to + 20%, which could be considered to be not significant (EC 2021), with the exception of MTCA in pig liver, IMT in pig kidney, and EBT in chicken muscle, for which the matrix effect ranged from − 25.9 to 28.7%. Without complicated purification to remove biological compound during the sample preparation step, the matrix effect of the developed method was an acceptable range in most cases.

### Application

The applicability of the developed method was evaluated by analyzing the l-tryptophan impurities in different meat products. Several kinds of commercially available meat products sold in a local supermarket and slaughterhouse in Korea were analyzed by using LC–MS/MS under the developed analytical method. As shown in Table 5, HTP, IMT and EBT were not detected in any sample tested. PIC and MTCA were detected in some of the samples. A very small amount of MTCA was detected in pig liver and kidney. PIC was detected in most of the samples, and relatively high levels of PIC were detected in organs such as the liver, kidney, and lung.

**Table 5:** Residue of the l-tryptophan impurities in commercial meat products

	Country of origin	HTP (μg/kg)	PIC (μg/kg)	MTCA (μg/kg)	IMT (μg/kg)	EBT (μg/kg)
Pig muscle	Republic of Korea	N.D.	46.6	N.D.	N.D.	N.D.
	Republic of Korea	N.D.	62.4	N.D.	N.D.	N.D.
	Republic of Korea	N.D.	38.5	N.D	N.D.	N.D.
	Spain	N.D.	115.5	N.D.	N.D.	N.D.
	United States	N.D.	227.7	N.D.	N.D.	N.D.
Pig skin	Republic of Korea	N.D.	30.5	N.D.	N.D.	N.D.
	Republic of Korea	N.D.	25.4	N.D.	N.D.	N.D.
Pig liver	Republic of Korea	N.D.	193.6	N.D.	N.D.	N.D.
	Republic of Korea	N.D.	441.2	5.3	N.D.	N.D.
Pig kidney	Republic of Korea	N.D.	356.5	N.D.	N.D.	N.D.
	Republic of Korea	N.D.	322.2	5.4.	N.D.	N.D.
Pig lung	Republic of Korea	N.D.	331.8	N.D.	N.D.	N.D.
	Republic of Korea	N.D.	447.6	N.D.	N.D.	N.D.
Chicken muscle	Republic of Korea	N.D.	55.0	N.D.	N.D.	N.D.
	Republic of Korea	N.D.	41.9	N.D.	N.D.	N.D.

*N.D.: not detected

Up to now, no analytical report regarding the l-tryptophan impurities that are related to EMS in meat products as well as food or feed product has been published. Developed method in this study is the first study that analyzes l-tryptophan impurities in the biological matrices. Furthermore, the analytical result indicated that the proposed method was effective and feasible to detect l-tryptophan impurities in meat products. However, there was no information available on how much l-tryptophan and l-tryptophan-derived impurities have been fed to pigs and chickens. Therefore, a further study is necessary to observe the residue of l-tryptophan impurities in meat products after feeding a known amount of l-tryptophan and l-tryptophan impurities to an animal.

## Conclusion

The safety of l-tryptophan has been under intense debate for several decades. The outbreak of EMS in 1989 was first considered to be a direct effect of l-tryptophan but was later determined to originate from impurities in the batches produced by a particular manufacturer. The causative substances of EMS-associated l-tryptophan were identified by several researchers. In this study, we developed an advanced analytical method for the detection of EMS-related l-tryptophan impurities in meat products.

Although the analytical method was developed to detect l-tryptophan impurities in meat matrices, we provided simple, faster and higher sensitivity compared to the previously reported method that used HPLC coupled with different kinds of detectors such as UV, florescence and amperometric detectors. Therefore, this method is applicable for the analysis of l-tryptophan impurities in meat matrices and is simple, sensitive, rapid, reliable, and accurate.

## Data Availability

The authors declare that the data supporting the findings of this study are available within the article.

## Abbreviations

AAA: (S)-2-Amino-3-(2-((S,E)-7-methylnon-1-en-1-yl)-1H-indol-3-yl) propanoic acid, or (S)-2-amino-3-(2-((E)-dec-1-en-1-yl)-1H-indol-3-yl) propanoic acid
EBT: 1,1′-Ethylidenebistryptophan
EMS: Eosinophilia-myalgia syndrome
HIT: 2-(2-Hydroxyindoline)-tryptophan
HPLC: High-performance liquid chromatography
HTP: (S)-2-amino-3-(5-hydroxy-1H-indol-3-yl)propanoic acid (5-hydroxytryptophan)
IMT: 2-(3-Indoylmethyl)-l-tryptophan
LC–MS/MS: Liquid chromatography—tandem mass spectrometry
LOQ: Limit of quantification
MDL: Method detection limit
MTCA: 1-Methyl-1,2,3,4-tetrahydro-β-carboline-3-carboxylic acid
PAA: 3-Phenylaminoalanine
PIC: 3A-hydroxy-1,2,3,3a,8,8a-hexahydropyrrolo-[2,3-b]-indole-2-carboxylic acid
PTFE: Polytetrafluoroethylene
SRM: Selected reaction monitoring
UHPLC: Ultra high-performance liquid chromatography
